# Invitro synergistic activity of lactic acid bacteria against multi-drug resistant staphylococci

**DOI:** 10.1186/s12906-019-2470-3

**Published:** 2019-03-19

**Authors:** Jinal Bhola, Rama Bhadekar

**Affiliations:** Department of Microbial Biotechnology, Rajiv Gandhi Institute of IT and Biotechnology, Bharati Vidyapeeth (Deemed to be) University, Katraj, Pune, 411046 India

**Keywords:** Antimicrobial activity, Bacteriocins, Lactic Acid Bacteria, MRSA, Multi-drug resistant, Synergism

## Abstract

**Background:**

Multi-drug resistance in microorganisms is a serious problem at national as well as at a global level. Many researches have suggested alternatives to antibiotics with minimal or no major side effects. LAB is one of the most human-friendly probiotic strains known to mankind from times immemorial. With the objective to deal with progressing antibiotic resistance among microorganisms, the present work demonstrates the inhibitory activity of LAB consortium against MDR clinical isolates.

**Methods:**

Total of nine hospital isolates of staphylococci were obtained and distinguished as *S.aureus* and coagulase-negative *Staphylococcus* (CoNS) based on their ability to ferment mannitol and form clumping with citrated plasma. All the test organisms were tested for antibiotic sensitivity with HiMedia (India) Octadisc Combi 92. Sets of *L .plantarum*, *L .acidophilus* and *L.casei var. rhamnosus* were prepared and tested against a standard culture of *S.aureus* NCIM 2129 by agar well diffusion method. To identify the primary source of substances responsible for inhibitory action, whole broth, cell-free supernatant, and cell lysate was prepared from the above-mentioned set. These were tested for their inhibitory action initially against standard *S.aureus* NCIM 2127, followed by clinical isolates.

**Results:**

The antibiotic sensitivity profile revealed that all clinical isolates were multi-drug resistant. The maximum inhibitory potential was seen in a combination of the three LAB in the ratio 1:1:1. Highest antagonistic activity was observed with whole broth and cell lysate of LAB consortium. In liquid broth assay, the cell lysate of LAB consortium astoundingly exhibited up to 85% inhibition of multi-drug resistant *Staphylococcus* isolates.

**Conclusions:**

Our results suggest antagonistic role of LAB metabolites against methicillin resistant staphylococci.

## Background

Extensive use of antibiotics, even to cure a common cold, has led to the heightened development of resistance towards a variety of drugs amongst pathogens all around the globe [1]. Staphylococci are Gram-positive, clump-forming, salt tolerant and often hemolytic pathogens, responsible for numerous infections like skin lesions, abscesses, osteomyelitis, endocarditis, furunculosis, urinary tract infections, toxic shock syndrome, and food poisoning. They are the leading cause of nosocomially acquired infections like catheter-associated bacteremia and necrotizing pneumonia [2]. *Staphylococcus* species can be classified as coagulase-positive staphylococci (CoPS), and the coagulase-negative staphylococci (CoNS). CoPS mainly represent *Staphylococcus aureus*, an opportunistic microorganism with many potential virulence factors like surface proteins and other agents that promote colonization of host tissues, inhibit phagocytosis and damage host tissues causing disease symptoms. CoNS represent diverse species of staphylococci, like *S. epidermis*, *S. haemolyticus*, *S. lugdunensis*, and *S. saprophyticus*, with normally fewer virulence factors but an ability to form biofilms on implanted devices causing severe issues [3].

Staphylococci are well known for their ability to become resistant to antibiotics [4, 5], inclusive of second and third line drugs [6, 7]. Methicillin-Resistant *Staphylococcus aureus* (MRSA) is a perilous group of the bacterial pathogen which combines virulence, antibiotic/ drug-resistance, and a heavy rate of transfer. These infectious pathogens not only do increase treatment cost tremendously but also contribute to increased mortality and morbidity rates [8]. Limited established treatment options exist for such invasive infections [9]. Multiple drug resistance in Staphylococci is a major and growing problem and is now subdivided into hospital-acquired MRSA (HA-MRSA) and community-acquired MRSA (CA-MRSA) [10]. The epidemiology of MRSA is constantly changing, resulting in a variation in drug-resistance patterns throughout regions and countries [11]. Vancomycin once represented the paragon to treat such invasive infections. However, an increase in reports of in vitro resistance to vancomycin and clinical failures with such invasive infection accentuated the need to develop alternative therapies for treatment [10–12].

Similarly, the prevalence of multi-drug resistant (MDR) strains of common bacterial pathogens is increasing worldwide [13, 14]. Although antibiotics are available for the treatment of these infections, because of their numerous adverse effects and development of resistant strains, there is an urgent need to search for alternatives to synthetic antibiotics [15]. Therefore, various approaches have been adopted to deal with the progressing multi-drug resistance among such pathogenic species. Treatment with selected probiotic strains is one such solution that is comparatively safe and stable as they do not increase the risk of multi-drug resistance of these pathogens [16, 17]. Most Lactic Acid Bacteria (LAB), despite their origin, have the potential to inhibit the growth of pathogens, including problematic antibiotic-resistant isolates due to their ability to produce several antimicrobial metabolites [18]. Many researchers have proven the inhibitory activity of different probiotic strains against such infectious pathogens [19–23].

The present study aimed to evaluate the in vitro antibacterial activity of an effective consortium of lactobacilli against MDR staphylococci.

## Methods

### Strains and culture conditions

Three *Lactobacillus* species, *Lactobacillus plantarum* NCIM 2374 (NCIB 6376), *Lactobacillus acidophilus* NCIM 2660 (ATCC 11975) and *Lactobacillus casei var. rhamnosus* NCIM 2364 (ATCC 7469), were collected from the National Collection of Industrial Microorganisms(NCIM) at the National Chemical Laboratory (NCL), Pune.

A standard strain of *Staphylococcus aureus* NCIM 2127 was collected from NCIM, NCL, Pune. Nine random clinical isolates of *Staphylococcus* species (labeled as A to I) were collected from the Microbiology Laboratory at the Bharati Hospital, Katraj, Pune, India.

For revival and maintenance of LAB, de Mann-Rogosa-Sharpe (MRS) medium (HiMedia, India) was used, while the clinical isolates were enriched with Brain Heart Infusion (BHI) agar (HiMedia, India). All these cultures were incubated overnight (about 18 h) at 37 °C at still and at shaking (120 rpm) conditions respectively.

Colony morphology and Gram reaction of the cultures were tested. Staphylococci strains were tested for their reactivity with mannitol and citrated plasma, as stated by Turner and Schwartz [24], to distinguish *S. aureus* from other *Staphylococcus* species.

### Substantiation of the multi-drug resistance

A standard *Staphylococcus aureus* NCIM 2127 (ATCC 9144) and clinically obtained *Staphylococcus* A to I were tested for their sensitivity towards methicillin and other antibiotics. The cultures were allowed to grow overnight in BHI broth at 37 °C. The overnight cultures of staphylococci were adjusted to 1.0 at OD_600_ and swabbed on Muller-Hilton agar plates. An octa-disc ring (OCTADISCS COMBI 92 HiMedia, India) containing the following antibiotics: Amikacin 30 μg (AK), Ciprofloxacin 5 μg (CIP), Gentamicin 10 μg (GEN), Ceftazidime 30 μg (CAZ), Cefepime 30 μg (CPM), Cefoxitin 30 μg (CX), Cefoxatime 30 μg (CTX) and Ceftriaxone 30 μg (CTR) was placed on each swabbed plate. The plates were incubated at 37 °C and were observed the next day for the zone of clearance. Zone diameters were measured according to Barry A. et al. [25]. and were compared with the standard measures given by the CLSI Performance Standards for Antimicrobial Susceptibility Testing [26].

### Antimicrobial activity

Antimicrobial activity of *Lactobacillus* strains was assayed to evaluate their ability to inhibit the clinical isolates of MDR microorganisms.

Seven sets of whole broth (WB) of *L. plantarum*, *L. acidophilus* and *L. casei var. rhamnosus*, individually as well as in combinations, were prepared as described as below to test their antimicrobial activity against the standard culture *S. aureus* NCIM 2127.

- I: *L. plantarum*
- II: *L. acidophilus*
- III: *L. casei*
- IV: *L. plantarum + L. acidophilus* (1:1)
- V: *L. acidophilus + L. casei* (1:1)
- VI: *L. plantarum + L. casei* (1:1)
- VII: *L. plantarum + L. acidophilus + L. casei* (1:1:1)

The activity was determined using the agar well diffusion method as described by Barbara et al., [27] with slight modification. For the preparation of each set, the culture broth of 0.5 O.D (McFarland Standard) for each culture was used. An overnight incubated culture of *S. aureus* was swabbed on 0.7% soft agar medium containing BHI+ MRS (1:1) (with 2% agar-agar base). Wells of 5 mm diameter were then punched on these pre-swabbed plates and 50 μl of each set was added in the wells accordingly. Inoculated sets were allowed for diffusion for 1 h at room temperature. The plates were then incubated overnight at 37 °C. Zone diameters were measured according to Barry A. et al. [25]. Inhibition was scored positive if the width of the clear zone around the well was observed.

The set giving the best result was used further to check the activity of WB, cell-free broth (CFB), and cell lysate (CL).

To prepare CFB, the set was centrifuged at 8000 rpm for 15 min at 4 °C. The supernatant was collected and passed through a 0.2-μm syringe filter (BioEra, India) to remove any remaining bacterial cells and cell debris. The cell lysate (CL) was prepared as per the protocol by Kang et al (2012). Cells separated from the above centrifugation were washed twice with saline and resuspended in lysis buffer (10 mM Tris HCl, pH 8.0; 1 mM EDTA; 0.1% (*w*/*v*) SDS). The sample was sonicated for 5–15 min until it appears milky. The sonicated sample was then centrifuged at 8000 rpm for 15 min at 4 °C and the supernatant was used for the experiment [28]. These samples ie. WB, CFB, and CL were used to investigate their activity against the clinical isolates by agar well diffusion method as described above.

To understand the antimicrobial activity of CL better, the liquid medium containing a mixture of LAB-CL with clinically obtained microorganisms was studied according to Barbara et al (2010) with slight modification. A 96-well ELISA plate was used for this purpose. 20 μl of each test organism (OD_600=_0.8) were mixed with 50 μl of CL. Each well was added with 130 μl of nutrient broth. Test microorganisms and CL individually were considered as controls. Optical Density at 595 nm was obtained with the help of Epoch microplate spectrophotometer (Biotek, USA).

All the experiments were performed in triplicates independently.

## Results

### Determination of coagulase positive and coagulase negative staphylococci

According to the MSA slant color change (reactivity with mannitol) and coagulase test, *Staphylococcus* isolates were distinguished to CoPS and CoNS. It was observed that *Staphylococcus* isolates A, B, C, D, E, and I were able to ferment mannitol, hence changing the MSA slant colour to yellow and showed agglutination when mixed with citrated plasma, hence may belong to *S.aureus* species; whereas *Staphylococcus* isolates F, G, and H neither showed any colour change on MSA nor any agglutination or clumping with the citrated plasma. It was also observed that *S.aureus* NCIM 2127, tested as a reference, was coagulase positive and was able to ferment mannitol.

### Substantiation of multi-drug resistance

The overnight incubated plates with antibiotic discs were observed and the zone diameters were measured. These zone diameters were compared with the CLSI standards [26] to obtain inferences (Table 1).

**Table 1:** Antibiotic susceptibility profile of clinical isolates and *S.aureus* NCIM 2127 (zone diameters achieved were compared with the CLSI standards)

Zone diameters in mm								
SAMPLES	AK	CIP	GEN	CAZ	CPM	CX	CTX	CTR
Staph A	12 (R)	11 (R)	7 (R)	17 (I)	15 (I)	17 (R)	24 (S)	16 (I)
Staph B	10 (R)	17 (I)	9 (R)	13 (R)	17 (I)	19 (R)	24 (S)	17 (I)
Staph C	11 (R)	10 (R)	8 (R)	17 (I)	17 (I)	19 (R)	24 (S)	16 (I)
Staph D	11 (R)	10 (R)	8 (R)	17 (I)	16 (I)	15 (R)	22 (I)	16 (I)
Staph E	10 (R)	17 (I)	10 (R)	16 (I)	17 (I)	15 (R)	21 (I)	14 (I)
Staph F	8 (R)	9 (R)	4 (R)	11 (R)	11 (R)	10 (R)	11 (R)	11 (R)
Staph G	10 (R)	11 (R)	11 (R)	14 (R)	12 (R)	14 (R)	12 (R)	11 (R)
Staph H	11 (R)	13 (R)	11 (R)	13 (R)	12 (R)	13 (R)	13 (R)	8 (R)
Staph I	11 (R)	11 (R)	8 (R)	13 (R)	16 (I)	19 (R)	17 (I)	16 (I)
*S.aureus* NCIM 2127	22 (S)	21 (S)	22 (S)	24 (S)	21 (S)	23 (S)	24 (S)	24 (S)

*S* sensitive, *I* intermediate, *R* resistant

Amikacin, Ak [R:< 14; I:15–16; S:> 17]; Ciprofloxacin, CIP [R:< 15; I:16–20; S:> 21]; Gentamicin, GEN [R:< 12; I:13–14; S:> 15]; Ceftazidime, CAZ [R:< 14; I:15–17; S:> 18]; Cefepime, CPM [R:< 14; I:15–17; S:> 18]; Cefoxitin, CX [R:< 21; S:> 22 (for CoPS) R:< 24; S:> 25 (for CoNS)]; Cefotaxime, CTX [R:< 14; I:15–22; S:> 23]; Ceftriaxone, CTR [R:< 13; I:14–20; S:> 21]

As a result of the antibiotic susceptibility test for the clinical isolates, all were observed to be resistant against gentamicin (GEN) and cefoxitin (CX). Among the other six antibiotics, amikacin (CTX) possessed the maximum inhibitory potential against the nine *Staphylococcus* strains. All the clinical isolates were MDR as they exhibited resistance towards at least three antibiotics belonging to different classes. *S. aureus* NCIM 2127 was observed to be sensitive towards all the antibiotics.

### Synergistic activity of *Lactobacillus* cultures

Agar well diffusion procedure was performed with seven sets as described above. The overnight incubated plates were observed and the zone diameters were measured (Table 2).

**Table 2:** Diameters of growth inhibition zones of *S.aureus* NCIM 2127 by individually prepared sets of *Lactobacillus sp.*

SETS	I	II	III	IV	V	VI	VII
Zone of inhibition (mm)	13.6	10.3	10.6	14.3	12.3	10.6	20.3
	±0.50	±0.47	±0.56	±0.30	±0.6	±0.18	±0.47

± − Standard deviation values derived from the mean of data from three independent experiments

It was observed that all the three strains of *Lactobacillus sp.* exhibited antimicrobial activity against *S.aureus*. The zone of inhibition was observed to be maximum with set VII ie. a consortium of all three LAB cultures with zone diameter of 20.3 ± 0.47 mm. This was followed by set IV ie. *L.plantarum* and *L. acidophilus* with a zone diameter of 14.3 ± 0.30.

### Inhibitory effect of selected LAB consortium

As discussed above, set VII showed the best activity in the agar well diffusion assay against *S. aureus* NCIM 2127. Hence, it was further used as WB, CFB and CL for comparative evaluation initially against *S. aureus* NCIM 2127 and then against all the clinical isolates. Data represented in Table 3 reveals the inhibitory action of actively growing cells, acid supernatant and cell lysate of the mixture of *Lactobacillus*.

**Table 3:** Diameters of growth inhibition zone of MDR microorganisms by WB, CFB, and CL

Samples	Zone of inhibition (mm)		
	WB	CFB	CL
*S.aureus* NCIM 2127	19.67 ± 0.06	10.34 ± 0.12	17.67 ± 0.25
Staph A	20.34 ± 0.014	9.34 ± 0.25	14.34 ± 0.26
Staph B	17.67 ± 0.40	8.34 ± 0.46	13 ± 0.60
Staph C	16.34 ± 0.5	8 ± 0.24	13.34 ± 0.12
Staph D	15 ± 0.10	8.34 ± 0.15	13.34 ± 0.42
Staph E	16 ± 0.12	7.64 ± 0.26	14 ± 0.15
Staph F	19.34 ± 0.37	10.34 ± 0.34	13.67 ± 0.12
Staph G	18.34 ± 0.15	10 ± 0.12	13.67 ± 0.12
Staph H	19 ± 0.26	8 ± 0.84	15 ± 0.34
Staph I	16.67 ± 0.12	6.67 ± 0.25	14 ± 0.26

±: Standard deviation values derived from data of three independent experiments

It was observed that WB, CFB as well as CL, all three exhibits antibacterial activity against *S.aureus*, MRSt as well as on MDR isolates.

The whole broth of set VII showed the maximum inhibitory effect on MRSt A to I and *S.aureus* NCIM 2127. Supernatant exhibited the least inhibitory effect on all the clinical isolates. On comparing with CFB, it was observed that CL had higher inhibitory activity against all the clinical isolates*.*

### Quantitative assay using LAB cell lysate

To understand the action of cell lysate of LAB against MDR organisms better, cultures were treated with cell lysate in liquid broth and checked for the change in optical density.

**Fig. 1:**
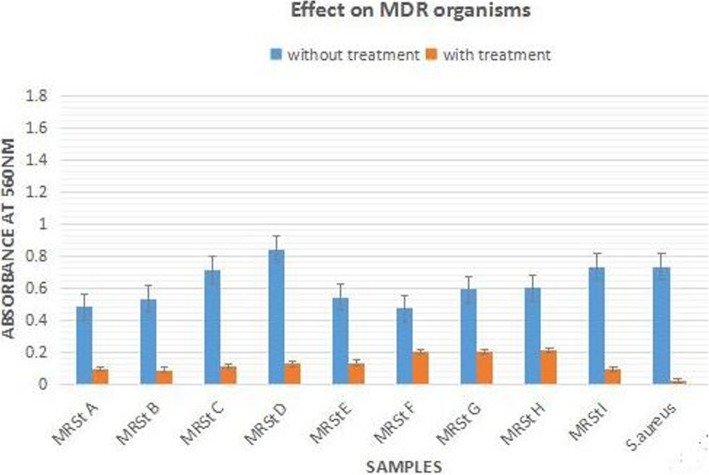
Growth of organisms measured in terms of absorbance at 560 nm when treated with cell lysate of selected LAB consortium. Error bars indicate standard deviation. *S.aureus NCIM 2127

It was observed (Fig. 1) that the standard culture of *S.aureus* NCIM 2127 showed the maximum ie. 96.6% inhibition. Amongst the MRSt, CoNS isolates (F, G, and H) showed inhibition in the range of 57.4–65%. The cell lysate was able to inhibit the MDR/MRSA isolates (A, B, C, D, E, and I) by an average of 83.1%.

## Discussion

The study revealed that from the nine clinical isolates of *Staphylococcus* species, six were coagulase positive and had an ability to ferment mannitol, hence belong to *S.aureus species*; whereas three were coagulase negative and were unable to ferment mannitol. Coagulase protein is an important virulent agent of *S. aureus* which can clot plasma into the gel [29]. However, there are reports available showing agglutination organisms other than *Staphylococcus* species [30].

A further confirmatory test is to find the ability of *S.aureus* to ferment mannitol. *S.aureus* ferments mannitol sugar and produce acid as an end product. Hence on inoculation with MSA, the color turns from pink to yellow [31]. CoNS do not ferment mannitol and hence the color of the slant remains pink [3]. CoNS include species other than *S. aureus*, like *S. epidermis*, *S. haemolyticus*, *S. lugdunensis,* and *S. saprophyticus* [32].

The antibiotic susceptibility assay revealed that the *Staphylococcus* isolates were resistant towards cefoxitin. Methicillin is a semisynthetic derivative of penicillin to treat penicillin-resistant *Staphylococcus* infections. Methicillin resistance is a marker of resistance to β lactam antibiotics (i.e., the penicillin and most cephalosporin antibiotics), which are some of the most commonly used antibiotics globally. Further, MRSA can easily develop resistance towards many other steroidal and non-steroidal antibiotics [33]. This means that even though methicillin is not used much these days, the resistance of bacteria towards it indicates drug resistance against multiple antimicrobial agents.

It is a well-known fact that microbes acquire multi-drug resistance due to various factors. Two most common mechanisms are (i) genetic mutations within the microorganism and (ii) triggering of mobile genetic elements that secure drug resistance genes [34]. Aminoglycoside (AG) antibiotics like amikacin and gentamicin are used to treat many Gram-negative and some Gram-positive infections. Gentamicin is the most used AG antibiotic while amikacin (semisynthetic AG) is usually prescribed less to prevent resistance development. Amikacin is persuasive towards most AG-resistant pathogens since it has a refracting nature towards most aminoglycoside-modifying enzymes. However, the upsurging resistance to amikacin has led to limitations of drugs for the treatment of infections in neonates [35, 36]. Research by Wenchang et al., [37] revealed that resistance development towards amikacin is associated with the thickening of the bacterial cell wall. However, resistance to AG drugs does not always mean resistance to other antibiotics. The strains used by the researcher showed susceptibility to antibiotics like ceftizoxime, chloramphenicol, ciprofloxacin, gentamicin, rifampicin, tetracycline, teicoplanin, linezolid, and vancomycin. The current work partially relates to these observations as the clinical isolates of MRSA are resistant towards amikacin and gentamicin. All of these clinical isolates can be considered as MDR as they are resistant towards antibiotics belonging to three different classes (aminoglycosides, quinolone and, cephalosporins) [38, 39].

The strains used in the study are *Lactobacillus plantarum* NCIM 2374 (NCIB 6376), *Lactobacillus acidophilus* NCIM 2660 (ATCC 11975) and *Lactobacillus casei var. rhamnosus* NCIM 2364 (ATCC 7469). All of these are industrially used strains and were selected with reference to the study concluded by Sikorska and Smoragiewicz [2013]. Their study concluded that most active strains against MRSA were *Lactobacillus reuteri*, *Lactobacillus rhamnosus GG, Propionibacterium freudenreichii*, *Propionibacterium acnes*, *Lactobacillus paracasei*, *L. acidophilus*, *L. casei*, *Lactobacillus plantarum*, *Lactobacillus bulgaricus*, *Lactobacillus fermentum* and *Lactococcus lactis*. Their effects were mediated both by direct cell competitive exclusion as well as the production of acids or bacteriocin-like inhibitors [40]. Similarly, many studies have been conducted using these LAB species to test their antimicrobial activity against MRSA [21, 27, 41].

Set VII showed maximum inhibition against *S.aureus*. These results demonstrate the synergistic effect of the combination of *L.plantarum*, *L.casei* and *L. acidophilus* (Set VII) in ratio 1:1:1, exhibiting highest antimicrobial activity as compared to the other sets.

It can be hypothesized that a combination of probiotic strains may complement each other’s effects or improve benefits or properties [42–44]. The objective of this study was, therefore, to determine if the chosen probiotics in the combinations tested may increase or enhance each other’s beneficial properties and their potential applications in favoring maximum inhibition against *S.aureus* and MDR staphylococci. The work can be co-related with research work by Karska-Wysocki [27] where the activity of *L. acidophilus* and *L. casei* in equal proportion (1:1) was observed to be higher when used in a combination.

To understand the primary action source of inhibition, the action of whole broth, cell-free supernatant and cell lysate were compared with each other. Generally, the LAB are the most implicated of the probiotic organisms, particularly those of the genera *Lactobacillus* and *Bifidobacterium*, which protects their territory by secreting acids like lactic, acetic, formic, succinic, glutamic, citric, and butyric acids; thereby creating an environment which is inhospitable to disease-causing bacteria. Lactobacilli are known to be microaerophilic by nature. They produce metabolites which can cause alterations in the oxidation-reduction potential, hence making the environment unfavorable for aerobic organisms. This action contributes to the overall inhibiting effect of these probiotic bacteria [45, 46]. Different *Lactobacillus* species have shown significant activities against classical antibiotic-resistant bacteria, MRSA and other emerging antibiotic resistant microorganisms. Voravuthikunchaia [47] stated that the antibacterial activity of the lactobacilli might cause growth inhibition and cell death with respect to the pathogen it is dealing with. Different mechanisms to exert antimicrobial effect are shown by the LAB, but the cell envelope is generally the target. Metabolic by-products such as bacteriocins, hydrogen peroxide (H_2_O_2_), and organic acids, produced by the lactobacilli during growth, contribute to their antibacterial activity. Other mechanisms proposed for their microbial antagonism are competition for nutrition, adhesion inhibition of pathogens to surfaces and stimulation of the immune system [47]. Studies have revealed that *L.reuteri*, isolated from a healthy vaginal ecosystem, [47] and *L.fermentum* [48]; can appreciably inhibit pathogens like MRSA. It has been observed that *L. casei* can displace and kill *S. aureus* adhering to human intestine mucus by 39 to 44% [49]. Charlier et al. (2008) reported that *L.lactis* had a specific antimicrobial activity against *S.aureus* [50]. Later, Koji (2005) stated the beneficial effects conferred by lactobacilli, including inhibition of Gram-negative and positive pathogenic bacteria [51].

Earlier, researchers have observed that the bacteriocins produced by LAB get adsorbed on the cell surface at specific pH [52–56]. Yang et al. [57], further discovered a technique for the purification of bacteriocins, responsible for the antimicrobial activity. In view of this, cell lysate of selected LAB consortium (present work) was prepared and studied further for their inhibitory action against MDR clinical isolates. The pH of 6.0–5.5 was observed after an overnight incubation, which is an ideal pH at which the proteins get adsorbed on the cells. Hence, the cell lysate contains both adsorbed proteins and intracellular proteins. This study revealed the potential of cell lysate of the combination of the LAB to inhibit MDR organisms. To our knowledge, antimicrobial activity by cell lysate is very less researched and present study gives an indication that the inhibitory activity of bacteriocins and some intracellular proteins of LAB can be of great importance.

## Conclusions

From all the in vitro testing, it can be clearly concluded that *S. aureus* was vulnerable to the metabolites produced by the LAB cultures selected, individually as well as in combinations. However, a combination of all the three strains used together exhibited the best result. This study showed the potential of the whole broth and cell lysate of the combination of *L.acidophillus, L. plantarum*, and *L. casei var. rhamnosus* as a better inhibitor towards MDR clinical isolates.

## Abbreviations

CFB: Cell-free broth(supernatant)
CL: Cell lysate
LAB: Lactic Acid Bacteria
MDR: Multi drug resistant
MRSA: Methicillin-Resistant *Staphylococcus aureus*
MRSt: Multi-drug resistant *Staphylococcus*
WB: Whole broth
